# Using Probability Estimates to Evaluate a Patient With Weakness

**DOI:** 10.7759/cureus.21775

**Published:** 2022-01-31

**Authors:** Alex Guzner, David Goese, Lawrence Yuen

**Affiliations:** 1 Internal Medicine, Northwestern University Feinberg School of Medicine, Chicago, USA; 2 Hospital Medicine, Northwestern University Feinberg School of Medicine, Chicago, USA

**Keywords:** probability estimates, probability estimation, polymyalgia rheumatica, spinal epidural abscess, diagnostic error, cognitive error, bias, weakness, back pain, superforecasting

## Abstract

In this case report, we review how probabilistic reasoning can be implemented in retrospect to refine the diagnostic process. A 67-year-old female with a history of polymyalgia rheumatica (PMR) and a recent dental procedure presented with weakness, falls, and chills ongoing for two weeks. She reported pain in her shoulders and lower back. On presentation, she was febrile, and labs were notable for leukocytosis with neutrophilic predominance and an elevated erythrocyte sedimentation rate (ESR). Chest radiograph revealed a left lower lung opacity, which was not seen on a repeat film. She was treated with antibiotics for community-acquired pneumonia and steroids for an exacerbation of PMR. After eight days of hospitalization, she was transferred to a subacute rehabilitation facility. A month later, she was readmitted with worsening lower back pain and right lower extremity weakness. Imaging revealed discitis and osteomyelitis at L1-L2. A spinal epidural abscess was present, leading to severe compression of the cauda equina nerve roots. Aspirate was positive for group B streptococcus. With antibiotic treatment alone, she recovered with resolution of her weakness.

In reviewing the literature, it becomes evident where improvements could have been made in the diagnostic process. Fever, leukocytosis, and neurological weakness are not commonly associated with PMR exacerbations. Lack of cough or shortness of breath, a persistently elevated erythrocyte sedimentation rate and C-reactive protein despite antibiotic treatment, and a repeat chest radiograph without an opacity suggest an alternative diagnosis to pneumonia. Persistent back pain with an insidious onset is a feature of untreated spinal epidural abscess. Steroid use and dental procedures are possible risk factors for spinal epidural abscess. By shedding light on how probabilities should be estimated, we hope to encourage probabilistic thinking to improve diagnostic accuracy. As with the best political forecasters, making precise probability estimates and frequently updating them may improve diagnostic accuracy for clinicians.

## Introduction

In political forecasting, making probability estimates and updating them were found to be associated with improved accuracy. As described in a book and a series of articles, Philip Tetlock recruited a group of forecasters who estimated the probabilities of various political events [1-3]. Determining a base rate, that is, a pre-test probability, and incorporating new data to derive an updated probability estimate, in other words, a post-test probability, were associated with a larger percentage of accurate predictions.

Establishing a pre-test probability provides a strong foundation to inform further probability estimates. Traditionally, it has been suggested that the pre-test probability should be derived from clinical experience or epidemiological studies [4]. Optimally, a pre-test probability should be derived from a study performed on a group of patients matching the patient seen in the clinical setting. While there may be challenges finding appropriate studies, a study suggested that research is available to guide estimates of pre-test probabilities for most patients admitted to an inpatient service [5]. 

Once a pre-test probability is obtained, further data can be applied to the pre-test probability in the form of likelihood ratios to derive a post-test probability. While likelihood ratios may not be available due to time constraints, a probability estimate of the change in probability based on new data may have to suffice. Under certain circumstances, reevaluation of probability estimates can improve diagnostic yield and reduce diagnostic errors. 

In this case report, we provide a retrospective review of a case report using probability estimates backed by clinical studies.

## Case presentation

A 67-year-old female with a history of polymyalgia rheumatica (PMR), chronic venous insufficiency, and recent root canal, presented to the emergency department with weakness of two weeks' duration. She reported pain in her shoulders and lower back. During this time, she had five falls and had been in two minor automobile accidents. She developed progressive weakness and eventual inability to get out of bed. Previously, she had been able to ambulate with a cane. 

**Figure 1:**
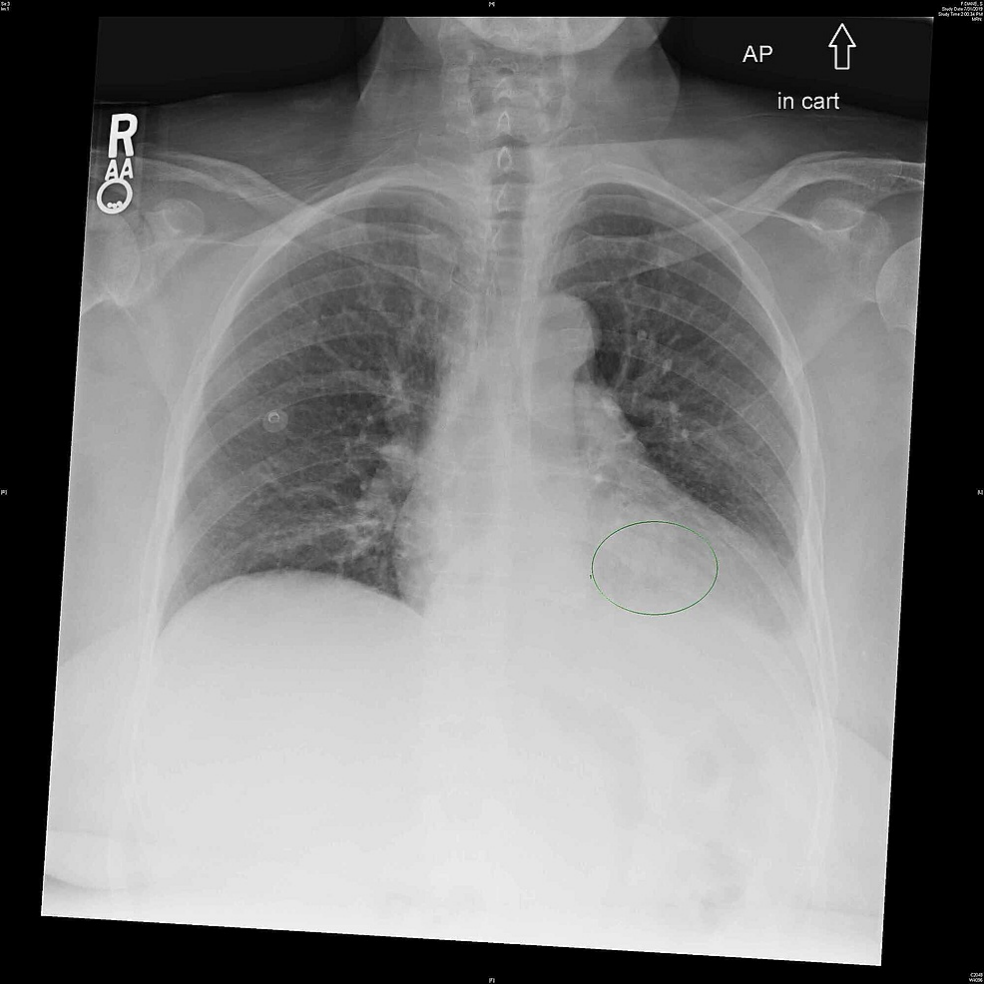
Chest radiograph was initially read as revealing a possible left lower lung opacity.

On presentation to the emergency department, she had a temperature of 38.4 degrees Celsius and a heart rate of 101 beats per minute. She appeared in no acute distress. Lung exam revealed coarse breath sounds on the left. The neurological exam was notable for 4/5 left grip strength. Left arm extension and flexion strength were 4/5. Evaluation of lower extremity strength, reflexes, and gait was not documented. White blood cell count was 27.8 K/uL (normal 3.5-10.5 K/uL) with 85% neutrophils, erythrocyte sedimentation rate (ESR) was 80 mm/hr (normal 4-25 mm/hr), and C-reactive protein (CRP) was 29.7 mg/L (normal 0.0-5.0 mg/L). Prior ESR around three months ago was 37 mm/hr and CRP was 32 mg/L. Urinalysis revealed no pyuria. Radiographs of the lumbar spine and pelvis were negative for any fractures. Chest radiograph was concerning for a left lower lung opacity (Figure 1). Ceftriaxone and azithromycin were started for community-acquired pneumonia. The patient was admitted to the general medicine service. 

**Figure 2:**
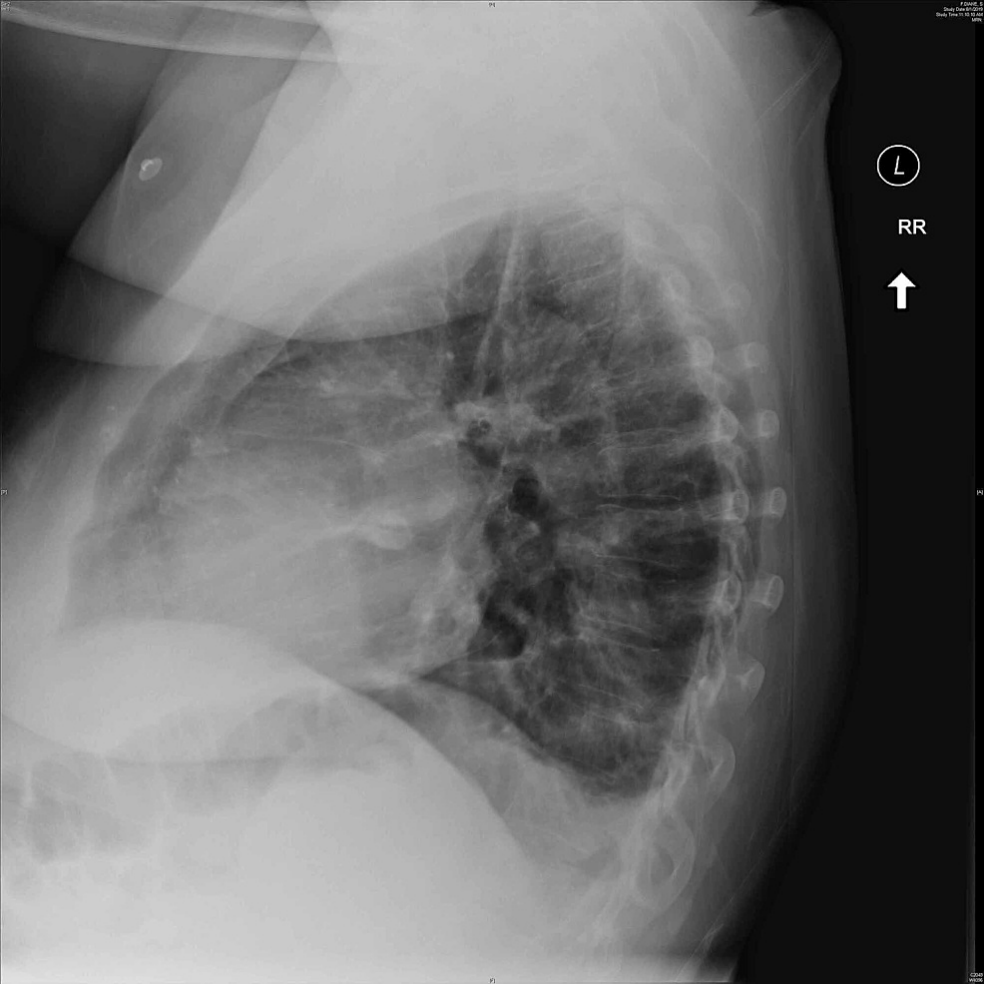
Subsequent lateral chest radiograph revealed no opacity.

The rheumatology service recommended prednisone 20 mg daily given concern for a recurrence of PMR. Posteroanterior and lateral chest radiographs two days later showed no lung opacity (Figure 2). Antibiotics were discontinued. Transthoracic echocardiogram did not reveal valvular vegetations. Blood cultures remained without growth. 

After five days of prednisone, the patient’s weakness had marginally improved with left greater than right lower extremity strength. A neurologic strength exam on day five of the admission documented 5/5 strength of bilateral hip extension, 2/5 strength of bilateral hip flexion, and 5/5 strength of bilateral foot plantarflexion and dorsiflexion. Reflex and sensory exams were not documented. On evaluation by the physical therapist, the patient was unable to transfer secondary to pain and hip flexion weakness. Repeat ESR was 92 mm/hr. CRP was 10.8 mg/dL. She was discharged to a subacute rehabilitation facility on daily prednisone until follow up with rheumatology for dose adjustment. 

**Figure 3:**
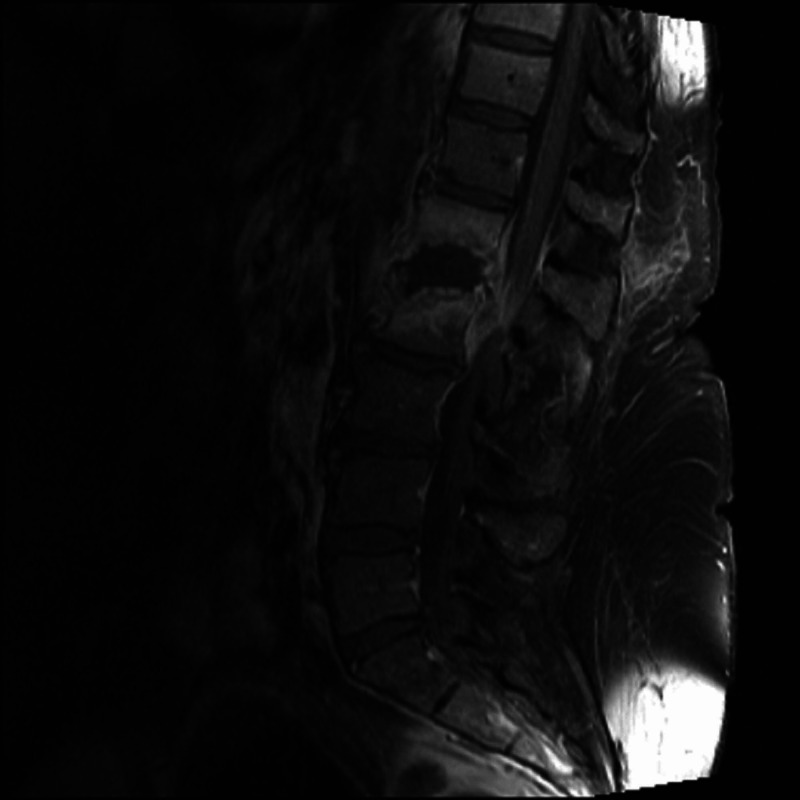
MRI revealed discitis and osteomyelitis at L1-L2 with spinal epidural abscess causing severe compression of the cauda equina nerve roots.

One month later, the patient returned to the emergency department with worsening right lower extremity weakness and inability to walk. A complete blood count and comprehensive metabolic panel were normal. MRI lumbar spine revealed osteomyelitis associated with a spinal epidural abscess leading to severe compression of the bilateral L1-L2 nerve roots (Figure 3). An associated phlegmon extended into the bilateral psoas muscles. Vancomycin and cefepime were initiated. Prednisone was discontinued. Neurosurgery recommended non-operative management. 16S rRNA sequencing performed on an aspirate obtained by interventional radiology was positive for Streptococcus agalactiae. She was subsequently transitioned to ceftriaxone. Her back pain improved over the next week. She was discharged to a skilled nursing facility with a six-week course of intravenous antibiotics. Following her course of antibiotics and physical therapy, she had further improvement of her back pain and was able to ambulate with a walker. 

## Discussion

The key characteristics of this patient’s initial presentation included fever, lower back pain, bilateral lower extremity weakness, shoulder pain, and unilateral upper extremity weakness. Tachycardia, leukocytosis, and fever suggested an infectious or inflammatory process. Empiric antibiotics were started for the initial diagnosis of community-acquired pneumonia, but were subsequently discontinued when posteroanterior and lateral films did not reveal an opacity. Concern shifted from an infectious etiology to the diagnosis of PMR. A full neurological exam was not initially performed. A limited neurological exam was performed five days into admission and demonstrated bilateral hip flexion weakness. 

The differential diagnosis at this point includes an exacerbation of PMR, community-acquired pneumonia, spinal epidural abscess, Guillain-Barré syndrome, and myasthenia gravis. In the following discussion, we will estimate the probability of these diagnoses and make adjustments based on clinical findings and diagnostic testing. 

Probability estimation occurs in the clinical setting based on factors including prior clinical experience, input from specialists, and data available from clinical studies. Deriving a range of probability estimates may be more appropriate than point estimate in certain situations. Widening or narrowing the range should be based on the strength of available literature or based on the level of certainty in comprehending a clinical situation. A wider range would suggest a lower degree of confidence. Another method to establish a range is to collect various probability estimates from colleagues after their review of the case and the literature. Time limitations make such a method impractical. At the least, in cases of diagnostic uncertainty when the range is noted to be wide, it signals the need for further data collection so that the range or probability estimates can be narrowed.

Establishing a range of probability estimates or a probability estimate down to the percentile decreases the risk of premature closure based on one data point. It permits updating of pre-test probabilities based on new data. Additionally, when one is unable to be precise in making a probability estimate and instead must use a range of probability estimates, uncertainty must be acknowledged. Lastly, establishing a probability estimate prior to the final diagnosis paves the way toward being able to provide quantitative feedback to one’s diagnostic process. Below, we will discuss the probability estimates relevant to this case with backing from studies and clinical reasoning.

Several key facts from a review of the literature decrease the probability of PMR as the correct diagnosis. White blood cell count above 12 K/UL was uncommon in patients with a diagnosis of PMR, occurring in 7.4% [6]. Fever above 38 degrees Celsius was present in only 12.5% of patients presenting with PMR according to a study reviewing 96 patients [7]. Recurrence of PMR was rare, occurring in only one of those 96 patients. Recurrence is differentiated from relapse, which occurs while a patient is still on steroids and recovering from an acute flare. The above clinical presentation would have represented a recurrence since she had previously completed a course of steroids for PMR. Additionally, the patient had weakness rather than stiffness. PMR is characterized by myalgias and stiffness of the proximal muscles. Neurological weakness is not a feature of PMR [8]. Furthermore, a rapid response to steroids is a characteristic feature of PMR and was not witnessed. Despite steroid administration in our patient, ESR remained elevated and pain persisted. The presence of fever, leukocytosis, back pain, and an incomplete response to steroids in conjunction with a low likelihood of recurrence in PMR reduce the probability estimate of this diagnosis to a low level. Calculating a precise post-test probability is not possible due to a lack of data as only small observational studies characterizing PMR are available. Nevertheless, based on the data presented, one may estimate a probability range of 1%-20% for the diagnosis of PMR.

On inspection of the literature, the probability of pneumonia as a cause of her symptoms was lower than had been considered. Respiratory symptoms commonly seen with pneumonia, such as cough or dyspnea, were not present. In one study, 96.5% of patients with community-acquired pneumonia presented with a cough [9]. Various studies have found insufficient data to calculate valid likelihood ratios for the absence of cough or dyspnea [10]. However, the absence of these symptoms lowers clinical suspicion for pneumonia as the etiology. The opacity that was seen on the portable radiograph was not detected on the lateral film two days later [11]. The providers caring for the patient subsequently reduced their probability estimate of pneumonia after which antibiotics were discontinued.

In evaluating the pre-test probability of various diagnoses, one must balance the likelihood of a given diagnosis with the potential consequences of missing it. Guillain-Barre Syndrome and myasthenia gravis are conditions that can cause extremity weakness and should be considered since if untreated they could lead to serious complications. Even so, a review of the literature demonstrates that the likelihood of these diagnoses in our patient is negligible. 

Based on the Brighton criteria, points are assigned to bilateral flaccid weakness and decreased deep tendon reflexes when attempting to diagnose Guillain-Barré syndrome [12]. In a 2014 study validating the Brighton Criteria, only one of 494 patients with Guillain-Barré syndrome had unilateral weakness [13]. Therefore, the likelihood of Guillain-Barré syndrome would remain low as the patient had unilateral upper extremity weakness. For the sake of assigning a probability estimate, we estimate the pre-test probability to be less than 1%.

Myasthenia gravis would also be an unlikely cause of our patient’s clinical presentation. A study of 152 patients with myasthenia gravis found only three patients (1.9%) who presented with weakness isolated to the limbs [14]. Without involvement of the neck, respiratory, bulbar, and ocular muscles, the likelihood of this diagnosis in our patient remains less than 1%.

Despite treatment with prednisone, the patient had persistent back pain and weakness of hip flexion. At this point in time, key clinical features conflicted with the leading diagnosis of PMR. When this type of conflict occurs, it represents an opportunity to reevaluate the situation using probabilistic reasoning. Alternative diagnoses should be considered. With ongoing back pain, leukocytosis, and persistent weakness of hip flexion despite treatment, the probability estimate of spinal epidural abscess as a cause of her symptoms increases. Bilateral weakness of hip flexion localizes specifically to the L1-L2 myotome, which increases concern for a focal neurological process.

The classic triad of epidural abscess consists of back pain, fever, and neurological deficits, although this triad is uncommon. For this reason, current literature advocates for a decision guideline based on the presence of back pain and risk factors that include intravenous drug use, contiguous or noncontiguous infection, diabetes, immunosuppression, steroid use, and recent instrumentation. Back pain associated with neurological weakness or fevers would warrant an emergency MRI. Otherwise, an erythrocyte sedimentation rate should be obtained if risk factors are present, and if abnormal, an MRI should be performed [15,16]. Alternatively, a pre-test probability can be estimated with application of likelihood ratios to derive a post-test probability. If the post-test probability rises above an established threshold, an MRI would be indicated (Table 1).

**Table 1:** 2 x 2 table used in estimating the post-test probability of a spinal epidural abscess based on an ESR > 50. Based on Table 1, a post-test probability was derived from an initial pre-test probability of 5% by conversion of the pre-test probability to a pre-test odds of 0.053. A positive likelihood ratio of 3.04 was calculated based on specificity and sensitivity. Applying the positive likelihood ratio to the pre-test odds led to a post-test odds of 0.161. Conversion to a post-test probability resulted in a probability of 13.8%. ESR = erythrocyte sedimentation rate; SEA = spinal epidural abscess.

	SEA (n=118)	No SEA (n=27)
ESR > 50	93	7
ESR < 50	25	20

While data remains lacking to calculate a precise pre-test probability in our patient, it can be argued that the patient’s fever and signs of lower extremity weakness increases the pre-test probability to at least 5%. This patient’s white blood cell count was initially 27.8 K/UL. ESR was significantly elevated at 80 mm/hr and eventually increased to 107 mm/hr despite treatment for both presumptive pneumonia and PMR. In one study, cases of spinal epidural abscess were matched to controls. An ESR above 50 had a positive likelihood ratio of 3.04. Applying a likelihood ratio of 3.04 to a pre-test probability of 5% results in a post-test probability of 13.8%. A white blood cell count above 11 has a positive likelihood ratio of 1.66. Applying a likelihood ratio of 1.66 to a pre-test probability of 5% results in a post-test probability of 8%. Given that the patient’s ESR and white blood cell count likely derive from the same inflammatory process, the actual post-test probability using these two factors alone is likely between 8% and 13.8%. With the post-test probability at 8% or higher for a potentially devastating diagnosis, an MRI would be indicated to rule out the diagnosis (Table 2).

**Table 2:** 2 x 2 table used in estimating the post-test probability of a spinal epidural abscess based on a WBC > 11. Based on Table 2, a post-test probability was derived from an initial pre-test probability of 5% by conversion of the pre-test probability to a pre-test odds of 0.053. A positive likelihood ratio of 1.66 was calculated based on specificity and sensitivity. Applying the positive likelihood ratio to the pre-test odds led to a post-test odds of 0.088. Conversion to a post-test probability resulted in a probability of 8.1%. ESR = erythrocyte sedimentation rate; SEA = spinal epidural abscess.

	SEA (n=162)	No SEA (n=87)
WBC > 11	108	35
WBC < 11	54	52

While prior dental work may raise concern for bacteremia with potentially seeding into the epidural space, the literature consists of case reports, which does not allow for the calculation of likelihood ratios [17,18]. 

Based on the above, we would assign a low initial probability estimate of approximately 5% for spinal epidural abscess. Subsequent factors such as a persistently elevated ESR may increase such an estimate to a range of between 10% and 15%. At this range, a threshold would have been reached where an MRI would have been strongly indicated.

## Conclusions

Diagnostic error remains prevalent due to the complexity of modern medicine. In our case study, we identify a missed diagnosis of spinal epidural abscess. The initial diagnosis of community-acquired pneumonia was abandoned and attributed to a false positive chest radiograph. There was an incomplete response to steroids, which decreased the likelihood of a PMR exacerbation. At discharge, there remained ongoing bilateral lower extremity weakness and back pain. At this juncture, there was an opportunity to exercise a clinical exercise in probability estimates. The time constraints of this exercise make it impractical to use for the care of every patient, but helpful in cases of diagnostic uncertainty. The act of probability estimation requires a pre-test probability from which to derive post-test probabilities based on new data. This process is used by the best political forecasters, who conduct frequent adjustments to further hone their probability estimates. Updating probability estimates is an invaluable diagnostic tool, which may lead to the avoidance of missed and delayed diagnoses.
